# Immune links in comorbid depression and psoriasis: A narrative mini-review and perspective

**DOI:** 10.1016/j.bbih.2025.100949

**Published:** 2025-01-15

**Authors:** Georgia Lada

**Affiliations:** a Dermatology Centre, Northern Care Alliance NHS Foundation Trust, Manchester Academic Health Science Centre, Division of Musculoskeletal & Dermatological Sciences, The University of Manchester, Greater Manchester M6 8HD, United Kingdom; b Greater Manchester Mental Health NHS Foundation Trust, Greater Manchester, United Kingdom

**Keywords:** Psoriasis, Depression, Brain-skin axis, Inflammation

## Abstract

Evidence suggests a bidirectional association between psoriasis and depression, which is considered to reflect complex neuroimmunological and psychosocial interactions. Despite an early interest in the brain-skin axis and the role of stress in psoriasis immunopathogenesis, there is ongoing limited preclinical and clinical research into the inflammatory links between depression and psoriasis. Existing findings for serum inflammatory markers of depression in psoriasis are inconsistent and do not fully align with those in the general population, while brain imaging evidence is scarce and has not confirmed direct brain involvement in the systemic inflammation of psoriasis. The present paper reviews the available literature on the immune interplay of psoriasis with depression, highlights the significance of further work in the field and proposes avenues for future research.

## Highlights

• Few data exist on neuroimmune contributions in the depression-psoriasis comorbidity.
• CRP findings are inconsistent and not well-aligned with the general population.
• Further, well-designed neuroinflammation studies can advance the field.
• PNI-informed mental health outcomes are needed in immunotherapy studies.
• Bench-to-bedside interdisciplinary collaboration is critical going forward.

## 1 Introduction

Although links of inflammatory skin disease with mood have been observed by clinicians since the 19th century (Willan, 1809), the “brain-skin axis” remains an underexplored landscape in the crossroads of psychiatry, immunology and dermatology. Psoriasis has been an early and major focus of brain-skin axis research (Seville, 1977). Psoriasis is a chronic, inflammatory skin disease which affects ∼1%–8.5% people globally (Parisi et al., 2013). Psoriatic inflammation usually manifests in red, scaly plaques, but also extends beyond the skin (Dowlatshahi et al., 2013). Depression is a common comorbidity of psoriasis with detrimental consequences for patients.

Inflammatory mechanisms are increasingly suggested to drive the association between psoriasis and depression, as systemic inflammation, including overlapping immune markers, are found in both conditions (Sforzini et al., 2023). Nevertheless, these mechanisms and their relevance for patient management remain largely obscure and few studies have directly examined whether the comorbidity is associated with the independently observed inflammation (Hölsken et al., 2021). Depressive symptom improvement following biologic treatments for psoriasis suggests downstream implications (Armstrong et al., 2024). Furthermore, a need for joint care of comorbid patients has been identified, however multidisciplinary psychodermatology clinics are scarce and psoriasis patients’ mental health needs remain unmet (Dalgard et al., 2018; Misery et al., 2023).

The present article first presents the epidemiological association between depression and psoriasis and highlights its significance for psychoneuroimmunological (PNI) research. It then discusses existing literature supporting immune psoriasis-depression links, focusing on evidence for systemic and brain inflammation underlying the comorbidity. Finally, it suggests directions for future research and clinical practice.

## 2 Methods

Pubmed/Medline and Google Scholar were searched from inception to August 01, 2024 using the following combination of terms: *psoriasis* AND *depression* AND (*inflammation* OR *cytokine* OR *chemokine* OR *interleukin* (IL) OR *tumour necrosis factor* (TNF) OR *C-reactive protein* (CRP) OR *T-cell* OR *neutrophil*). References of identified brain studies were screened. For the main synthesis in sections 5, 6, I considered original research which examines depression in conjunction with psoriasis and investigates systemic or brain inflammation. For narrative completeness, I also outline key evidence from the wider depression-centred and psoriasis literature supporting overlapping neural-immune pathways and markers. The present review is not exhaustive. Compared to systematic reviews, narrative reviews have the limitation of reflecting the author's perspective and potential article selection bias. Nevertheless, the present review aligns with and extends an earlier systematic review (Fabrazzo et al., 2021). Furthermore, it is relevant and adds to the literature as it primarily aims to (i) review research that directly investigates inflammation associated with the comorbidity, (ii) highlight the relevance of psoriasis-depression research for the wider PNI and (iii) suggest directions moving forward.

## 3 Significance of understanding the depression-psoriasis comorbidity and its inflammatory drivers

According to meta-analytical data, up to 19% of patients with psoriasis meet clinical depression criteria and one in four exhibit depressive symptoms (Dowlatshahi et al., 2014). It is well-replicated that psoriasis increases the risk for depression (Egeberg et al., 2018; Kurd et al., 2010). Furthermore, large-scale cohort studies suggest increased psoriasis incidence among depressed individuals (Dominguez et al., 2013; Min et al., 2020). In a prospective female nurse cohort, depressed women had higher relative risk for psoriasis (1.59 (95% CI 1.21–2.08)) (Dominguez et al., 2013). Conversely, in a study of >60,000 participants with depression, antidepressant treatment reduced psoriasis incidence independently of cardiovascular disease and obesity (Tzeng et al., 2021). This two-way association cannot be explained by psychosocial factors only; although psoriasis-associated internalised stigma undeniably affects mood (Rapp et al., 1999). Understanding biological depression drivers in psoriasis has clinical significance for both conditions. Depression worsens psoriasis-related quality of life (Fortune et al., 2005)and heightens patients' risk for cardiovascular disease (Egeberg et al., 2016) and psoriatic arthritis (PsA) (Lewinson et al., 2017). PsA is an inflammatory arthropathy affecting up to 30% of psoriasis patients and involves the spine, peripheral joints or entheses (Haberman et al., 2018). Comorbid PsA is considered linked not only to significant disability, but also to a higher systemic inflammation load compared to psoriasis only (Eder et al., 2013). Furthermore, anti-cytokine treatments for psoriasis are found to improve patients’ mood in both real-world studies and randomised controlled trials (RCTs) (Strober et al., 2018).

There are wider PNI implications of investigating biological depression-psoriasis links. The heterogeneity in depression manifestations and pathophysiology renders depression management challenging, and the immune system appears to be involved to a different extent and possibly in different ways across subpopulations with depression (Milaneschi et al., 2020; Pariante, 2021). As depressed populations sharing a manifest inflammatory condition are expected to have more homogeneous immunometabolical profiles, understanding the interplay of comorbidities in these populations is critical towards precision psychiatry. Notably, in a multi-centre European study, psoriasis had one of the highest depression rates and the highest suicidal ideation rate amongst ten most common skin diseases (Dalgard et al., 2015). Furthermore, these patients may more likely benefit from optimised immune-modulating treatments for their mood. Clinical trials in depressed people without inflammatory disease suggest that anti-inflammatory drugs improve depression among subgroups with (low-grade) systemic inflammation but not those without (Nettis et al., 2021; Raison et al., 2013).

**Fig. 1:**
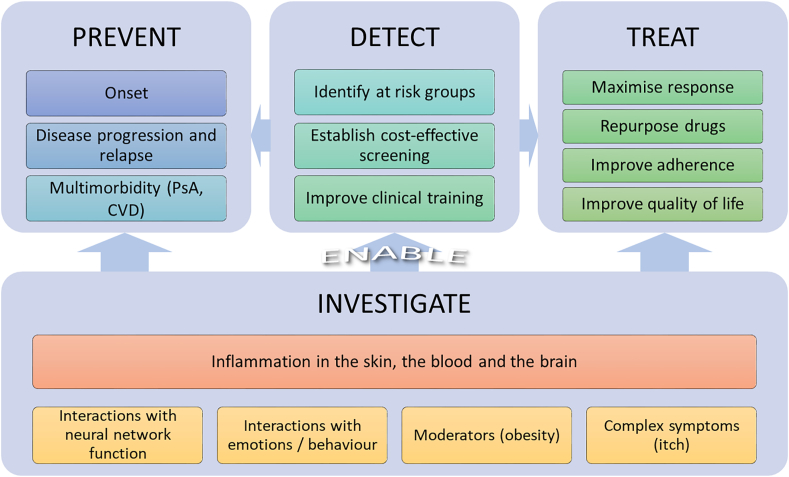
A schematic overview of potential research interest areas and clinical significance of understanding immune interactions between depression and psoriasis. PsA = psoriatic arthritis; CVD = cardiovascular disease. Identifying inflammatory signatures of the comorbidity at the molecular, cellular and tissue level both in the periphery and the brain and their interactions with neural network function, psychopathology, as well as potential complex modulating factors such as itch and obesity, could help develop cost-effective screening and early interventions targeting at risk groups and inform clinician training. Ultimate aims are to reduce psoriasis and depression morbidity at the general population level; prevent relapse, disease severity and multimorbidity in patients; maximise response to both immunomodulators and antidepressants; improve adherence through better drug effectiveness and depression control; reduce psoriasis- and depression-related disability; and explore drug repurposing for wider immunopsychiatry.

Psoriasis therefore constitutes a unique paradigm within which brain interactions with chronic inflammation can be studied (Kleyn et al., 2020; Peters et al., 2012). The psoriasis-associated “brain-skin axis” can serve as a starting point to understand wider interactions (“gut-skin-brain” axis), identify immune targets potentially useful in other populations with depression, and explore repurposing of immune-modulating drugs already used for people's skin (psoriasis) and/or joints (PsA) (Fig. 1).

## 4 Neuro-immune signalling in psoriasis and depression: brief overview

In psoriasis, skin lesions arise from complex processes involving maladaptive immune signalling among keratinocytes, dendritic cells, neutrophils and T-cells; implicated pathways include IL-17A, IL-22, IL-23 and TNF-α signalling (Albanesi et al., 2018; Koo et al., 2017). Inflammation extends in a systemic “march of psoriasis”, considered to manifest in comorbidities, including PsA, obesity and cardiovascular disease (Korman, 2020); its role for depression is unclear.

Brain-skin interactions in psoriasis and depression are considered to involve brain-to-immune and immune-to-brain signalling, which may be triggered by stress and be mediated by neuroendocrine systems.

Evidence from stress research in psoriasis suggests an important role of brain-to-immune-to-skin signalling. Psychological stress can trigger psoriasis onset and high daily stress predicts higher disease severity after one month (Verhoeven et al., 2009b). Worry appears to increase patients’ vulnerability to stressors and correlates with worse disease outcomes under high stress (Verhoeven et al., 2009a). The sympathetic nervous system (SNS) and the hypothalamic–pituitary–adrenal axis (HPAA) may mediate these effects.

An early controlled study found pronounced sympathetic reactivity in psoriasis, as suggested by increased stress-induced urinary adrenaline secretion (Arnetz et al., 1985). Buske-Kirschbaum et al. also showed greater plasma adrenaline and noradrenaline elevation as well as monocyte and CD4+cell counts increases in patients with psoriasis compared to healthy controls following a stress test (Buske-Kirschbaum et al., 2006, 2007). Although their sample was small (n = 48), these findings are important, as CD4^+^ cells and monocytes participate in skin infiltration preceding plaque formation in psoriasis. Nevertheless, studies investigating other SNS markers (e.g. α-amylase (De Brouwer et al., 2014); pulse rate (Richards et al., 2005)) have not found sympathetic dysfunction in psoriasis. SNS may exert complex effects and a HPAA-SNS coordination deficit has been suggested in psoriasis, as HPAA and SNS stress reactivity markers were shown to correlate poorly in patients in contrast to healthy controls (Richards et al., 2005). In depression, findings regarding SNS function are equivocal, however autonomic dysfunction correlates with serum inflammation in cardiovascular-associated depression (Li et al., 2024).

HPAA may also mediate psoriatic inflammation, although results are conflicting. In contrast to earlier studies showing no altered HPAA response in psoriasis post-stress (Buske-Kirschbaum et al., 2006) and following CRH stimulation (Karanikas et al., 2007), an experimental social stress paradigm found higher salivary cortisol increases in patients compared to controls (De Brouwer et al., 2014). However, HPAA activity may depend on disease characteristics. Richards et al. showed a hypocortisolaemic response to acute social stress in self-reported stress-reactive psoriasis compared to non-stress-reactive disease (Richards et al., 2005). Aligning with these results, Evers et al. found lower serum cortisol in chronically high-stress psoriasis patients compared to patients experiencing lower everyday stress; cortisol also negatively correlated with peak daily stress (Evers et al., 2010). HPA hypoactivity, which is also found in other chronic inflammatory diseases (Tsigos and Chrousos, 2002), may therefore characterise a patient subgroup vulnerable to stress and suggest adaptation of the glucocorticoid and inflammatory response to chronic stress (Pariante, 2017). HPAA-mediated stress responses in humans can induce mast cell activation, up- or down-regulate pro-inflammatory cytokine expression and impair the skin barrier function (Altemus et al., 2001; Arck et al., 2006). Cytokines interact with the HPAA at all levels and can also directly induce glucocorticoid resistance. Furthermore, the skin has its own HPA axis, which may modulate central responses; its dysfunction, including impaired cortisol synthesis and glucocorticoid receptor expression, has been suggested in psoriasis (Hannen et al., 2017). In depression, while the HPA axis is generally considered hyperactive, recent evidence shows more complex dysfunction, likely dependent on glucocorticoid resistance (Maes and Carvalho, 2018). Interestingly, in depressed patients with cardiovascular disease, a higher inflammatory load coexists with HPAA hypoactivity and glucocorticoid resistance, which may reflect a state of chronic stress and inflammatory activation (Nikkheslat et al., 2015).

Neuropeptides, notably substance P (SP), further mediate the cross-talk between nerves and skin cells. SP exacerbates stress-associated skin inflammation, inducing mast cell cytokine secretion (Harvima et al., 2008), and is abundant both in psoriatic lesions (Peters et al., 2012) and the CSF of depressed individuals (Geracioti et al., 2006). Its contribution into the depression-psoriasis comorbidity is unclear.

Little is known about immune-to-brain signalling in psoriasis. In depression, potentially implicated cytokine signalling pathways involve cytokines crossing the blood-brain-barrier (BBB) with the help of transport systems, passing through the circumventricular organs, or triggering BBB cells to release pro-inflammatory molecules in the brain; peripheral cytokines triggering vagal signal transmission; or activating monocyte recruitment into the brain (Capuron and Miller, 2011; D'Mello et al., 2009). This signalling can result into microglial activation and, in turn, disturbed neurotransmission, disrupted synaptic plasticity and ultimately inflammation-driven atrophy, including brain-derived neurotrophic factor (BDNF) reduction (Miller et al., 2009).

## 5 Evidence for systemic inflammation underlying the depression-psoriasis comorbidity

Meta-analyses of systemic inflammatory markers conducted separately in psoriasis (Dowlatshahi et al., 2013) and depression (Köhler et al., 2017; Osimo et al., 2020) show cytokine and acute-phase protein overlap, with serum CRP, IL-6, TNF-α and IL-18 elevation in both disorders. T-helper 17 (Th17) cells are considered key in psoriatic inflammation, and serum IL-17 levels mirror disease severity (Yang et al., 2024). Inflammation in psoriasis is sustained by neutrophil degranulation impairment (Nakabo et al., 2021) and increased plasma neutrophil activation markers are found (Rocha-Pereira et al., 2004). Also in depression, a meta-analysis showed raised neutrophils, monocytes and CD4^+^ T cells, although research on Th17 cells is limited (Foley et al., 2023). Neutrophil increases may primarily distinguish depressed patients’ immune cell profiles from those of non-depressed individuals (Lynall et al., 2020). Furthermore, serum BDNF decrease is found in both depression and depression-free psoriasis (Brunoni et al., 2015).

However, few studies have measured serum inflammatory markers of depression in psoriasis and most of them have examined CRP. Serum CRP elevation is a commonly used depression marker in the general population and shows independence of lifestyle factors (Pitharouli et al., 2021). In a sample with psoriasis (n = 53), Breuer et al. found an association between CRP and chronic stress in women, but not men (Breuer et al., 2016). Further research supports wider sex-specific CRP-depression links for women, which may be genetic- or hormone-driven (Derry et al., 2015), although findings are not unanimous (Silva et al., 2023). In a tertiary sample (n = 239), serum CRP correlated with depression scores after controlling for sex and psoriasis severity (Tan et al., 2023). In a UK Biobank sample of 5485 psoriasis patients, we did not find associations of CRP with depression in either sex; although this population had overall mild psoriasis (Lada et al., 2023).

In contrast, we discovered associations of increased neutrophil counts with historical depression in both sexes and with concurrent depressive scores among women with psoriasis (Lada et al., 2023). Associations were independent of lifestyle factors, psoriasis severity and comorbidity and would support possible neutrophil-associated pathways contributing to immune psoriasis-depression links, in particular for women. Furthermore, although serum IL-6 increase is robustly replicated in depression (Köhler et al., 2017), serum IL-6 did not correlate with depression in a study of men with psoriasis; however potential confounders were not adjusted for (Pietrzak et al., 2018).

It is not clear why CRP-depression associations are less consistent in psoriasis than in the general population. Physical comorbidities could mask depression contributions to inflammation (Leighton et al., 2018) and depressed patients prone to immune activation may not be reliably identified based on CRP (Sforzini et al., 2023). In psoriasis, obesity, psoriasis severity and PsA may moderate inflammation marker levels (Beygi et al., 2014) and their associations with depression. For example, in PsA, depression has been associated with serum IL-6, but not IL-17A or CRP (De Lorenzis et al., 2021; Lai et al., 2022).

Finally, antidepressant effects of anti-cytokine agents in psoriasis RCTs may indirectly support the hypothesis of immune-driven comorbid depression, however few studies have examined the role of inflammation in this effect. In three ixekizumab trials in psoriasis, depression remitted in up to 45% of comorbid patients following IL-17A treatment, however mood improvement was only weakly associated with CRP reduction (r = 0.11, p < 0.001) (Griffiths et al., 2017). In an early TNF inhibitor (TNFI) trial, depression improvement was not correlated with objective improvement in skin signs (Tyring et al., 2006). TNFIs were found to downregulate the serotonin transporter and increase serotonergic signalling in psoriasis (Krishnadas et al., 2016), which may contribute to their antidepressant effects. Furthermore, mediation analysis of comparator guselkumab trials showed that up to ∼50% of depression improvement under anti-IL-23 treatment, compared to TNFI adalimumab, is independent of skin improvement in psoriasis (Armstrong et al., 2024). This post-hoc finding suggests contributing biological anti-IL-23 effects, either directly on mood, or on systemic, potentially affectively modulating, psoriasis-associated symptoms (i.e. itch, sleep, fatigue).

To answer the question of whether biologics improve depression in psoriasis primarily due to improved psychosocial functioning following skin clearance, or via direct immunological benefits for the brain (which may be asynchronous to skin or systemic gains), it is important to understand whether there is immune activation within the psoriatic brain.

## 6 Evidence from brain studies

Growing evidence indicates brain inflammation in depression in the form of heightened translocator protein (TSPO) expression, and increased IL-6 and TNF-α in the cerebrospinal fluid (CSF) (Enache et al., 2019). In a sample with moderate-to-severe psoriasis without depression, neuroinflammation was not detected using TSPO positron emission tomography (PET) (Hunter et al., 2016). However, this sample (n = 12 patients) had relatively low serum inflammation levels, and its neuroimmunological profile may differ to patients most vulnerable to depression. Furthermore, TSPO PET markers may not reflect well systemic inflammatory processes (Enache et al., 2019).

To my knowledge, no clinical studies have investigated neuroinflammation in psoriasis in the presence of depression, using PET imaging, CSF markers or postmortem immunohistochemistry. Pre-clinical evidence is limited. In a imiquimod psoriasis model, psoriatic inflammation increased brain IL-17A levels; administration of both peripheral anti-IL17A agents and NFκB and p38MAPK inhibitors decreased brain cytokines as well as IL17A-induced depression-like behaviours (Nadeem et al., 2017). In a mouse model of psoriasis with depression/anxiety, fluoxetine ameliorated depression/anxiety, increased brain BDNF mRNA levels and improved psoriasis lesions. A BDNF receptor (TrkB) antagonist reversed these effects (JiaWen et al., 2018). BDNF-TrkB signalling may mediate neuroinflammation; for example, BDNF inhibits lipopolysaccharide-induced microglial activation (Wu et al., 2020).

A body of research has lately shifted towards magnetic resonance imaging (MRI) neuroinflammation markers, in part due to their translational potential and PET limitations. These consist of traditional structural and functional MRI metrics, diffusion MRI techniques, and advanced contrast agents, including BBB permeability markers (Oestreich and O'Sullivan, 2022).

The few existing MRI-derived brain data in populations with psoriasis point to an absence of atrophic changes in patients (Lada et al., 2022; Pezzolo et al., 2021). However, structural connectivity increases were shown in several white matter pathways in fourteen depression-free psoriasis patients (Najafi et al., 2020). Non depression-associated, resting-state functional connectivity changes have also been found in default mode network (DMN) regions in psoriasis (Yi et al., 2024). These studies did not examine systemic inflammation though. Using a sex- and age-matched UK Biobank sample (n = 1048), we investigated associations between depression and systemic inflammation in psoriasis and detected thickening of the right precuneus, a core region of the DMN (Dadario and Sughrue, 2023), in depressed patients with psoriasis compared to people with depression in the general population (Lada et al., 2022). However, this cortical change was not associated with serum CRP or neutrophil levels.

Overall, the limited existing research does not suggest direct brain immune activation or (neuro)inflammation-related scarring in psoriasis. However, given the research paucity, methodological limitations, and lack of evidence for core inflammatory cytokines implicated in psoriasis, an underlying brain inflammatory activity, not mirrored in non-specific serum markers, is not implausible. DMN/precuneus activation characterises itch (Papoiu et al., 2012; Yi et al., 2024), and existing findings may indicate affective modulation of psoriasis-related somatosensory processing. However, DMN connectivity disruption is also linked to inflamed depression (Kitzbichler et al., 2021; Marsland et al., 2017), and imaging DMN regions in relation to a wider range of immune markers in psoriasis would be important. It remains unclear whether any brain findings are unique to psoriasis or also characterise depression in other inflammatory skin diseases because of itch-related neuro-immune interactions.

## 7 Future directions

The above sections highlight a large number of unknowns in the immune relationship of psoriasis with depression and have identified some avenues warranting further investigation. Importantly, the field lacks much needed studies that use direct measures of brain inflammation in comorbid patients and appropriate controls, in conjunction with a larger array of serum markers, starting with cytokines targeted by biologics effective for psoriasis (such as IL-17, IL-22, IL-23). Longitudinal designs and sex stratification are paramount in understanding these complex relationships.

Parallel development of basic research as well as transcriptional studies are important to identify treatment targets and develop more specific markers for this cohort. Methylation changes in the psoriasis susceptibility *PSORSC1C3* gene promoter and other psoriasis-associated immune-regulating loci have been found in depressed individuals (Lapsley et al., 2020; Murphy et al., 2017), but it is unclear how they translate into the transcriptional profile of this comorbidity. Neutrophil-associated signatures of comorbid depression and psoriasis are worth exploring on the molecular level, in particular since circulating dysfunctional neutrophils in psoriasis appear to respond to biologics (Rodriguez-Rosales et al., 2021).

Randomised comparative trials of biologics targeting different immune axes are needed to guide treatment optimisation for patients with psoriasis and depression. Introducing PNI-informed, standardised mental health outcomes in these RCTs as well as psoriasis immunomodulator registries is critical. Increasing efforts are made to measure depression using clinical classification systems. It is also important to adopt a dimensional, even transdiagnostic, approach where possible. For example, the Hospital Anxiety and Depression Scale (Zigmond and Snaith, 1983) is the most commonly used depression questionnaire in psoriasis (Dowlatshahi et al., 2014), partly to overcome the issue of overlapping somatic symptoms, however it does not assess suicidality. By not capturing suicidality and somatic symptoms, potential inflammation-driven clinical targets in depression (Chu et al., 2019; Frank et al., 2021) may be missed.

Overarching such efforts should be interspecialty bench-to-bedside collaboration and knowledge sharing. Depression is unrecognised in over half of psoriasis patients (Dalgard et al., 2018), and may be erroneously normalised by both patients and clinicians (Bewley et al., 2013; Coventry et al., 2011). Although its immune drivers remain obscure, the psoriasis-depression comorbidity appears bio-psychosocial and cannot be adequately addressed if not acknowledged as such. Further investigations into the biology of their depression, along with systematic mental health screening and timely biopsychosocial interventions could ultimately improve these patients’ care. Training of psychiatrists and dermatologists to navigate clinical complexities with a PNI awareness as well as specialised psychodermatological services seem to be the way forward.

## Funding disclosure

This research was supported by the 10.13039/100014653NIHR Manchester Biomedical Research Centre Funding Scheme. The views expressed in this publication are those of the author and not necessarily those of the NHS, the NIHR or the Department of Health.

## Declaration of competing interest

The authors declare the following financial interests/personal relationships which may be considered as potential competing interests: G Lada has received speaker honoraria from Janssen, Lilly, Leo, and Novartis.

## Data Availability

This is a review article.
